# Vaccine hesitancy and behavior change theory-based social media interventions: a systematic review

**DOI:** 10.1093/tbm/ibab148

**Published:** 2021-11-29

**Authors:** Lan Li, Caroline E Wood, Patty Kostkova

**Affiliations:** UCL Centre for Digital Public Health in Emergencies (dPHE), Institute for Risk and Disaster Reduction, University College London (UCL), London, UK

**Keywords:** Vaccine hesitancy, Behavior change theory, Social media, Digital health intervention

## Abstract

It is widely acknowledged that vaccine hesitancy is a multifaceted problem that cannot be addressed by a single strategy. Behavior change theories and social media tools may together help to guide the design of interventions aimed at improving vaccination uptake. This systematic review aims to identify the breadth and effectiveness of such theories and tools. The systematic review search was performed in PubMed, Scopus, ACM, Cochrane Library, ProQuest, and Web of Science databases for studies between January 2011 and January 2021 that applied social media tools to increase vaccine confidence or improve vaccination uptake. The literature search yielded a total of 3,065 publications. Twenty articles met the eligibility criteria, 12 of which were theory-based interventions. The result shows that the Health Belief Model was the most frequently deployed theory, and the most common social media tool was educational posts, followed by dialogue-based groups, interactive websites, and personal reminders. Theory-based interventions were generally more measurable and comparable and had more evidence to trigger the positive behavior change. Fifteen studies reported the effectiveness in knowledge gain, intention increase, or behavior change. Educational messages were proved to be effective in increasing knowledge but less helpful in triggering behavior change. Dialogue-based social media intervention performed well in improving people’s intention to vaccinate. Interventions informed by behavior change theory and delivered via social media platforms offer an important opportunity for addressing vaccine hesitancy. This review highlights the need to use a multitheory framework and tailoring social media interventions to the specific circumstances and needs of the target audience in future interventions. The results and insights gained from this review will be of assistance to future studies.

## Teaser

The review identified the breadth and effectiveness of behavior change theories and social media tools used in interventions aimed at addressing vaccine hesitancy (the delay in acceptance or refusal of vaccination despite the availability of vaccination services). It highlighted the importance of using a multi-theory framework and of tailoring social media interventions to specific circumstances.

**Implications**

**Practice:** Findings of the review provide guidance for future interventions informed by behavior change theory and delivered via social media platforms, which could offer an important opportunity for addressing vaccine hesitancy.

**Policy:** Interventions using social media informed by behavior change theories provide a platform for low-cost and highly efficient policy advocacy opportunities for addressing vaccine hesitancy and increase vaccine confidence.

**Research:** The review builds on current literature to provide greater clarity on how social media and behavior change theories have been used to address vaccine hesitancy and increase vaccine confidence, and highlights the need for studies to evaluate interventions to address hesitancy and explore the potential of multitheory frameworks to inform the design of interventions.

## INTRODUCTION

Health threats caused by infectious diseases can result in significant and widespread increases in morbidity and mortality and enormous economic losses, and the mortality impact is greater in low- and middle-income countries. Vaccination is the most effective way to save lives from infectious diseases. Since the middle of the 20th century, vaccination campaigns have helped eradicate smallpox and have countered the fear of other deadly infectious diseases, such as polio, measles, and invasive pneumococcal disease [1]. Achieving adequate vaccine coverage can be threatened by people’s concerns about a particular vaccine or vaccination in general. Such concerns are known as *vaccine hesitancy*.

**Fig 1:**
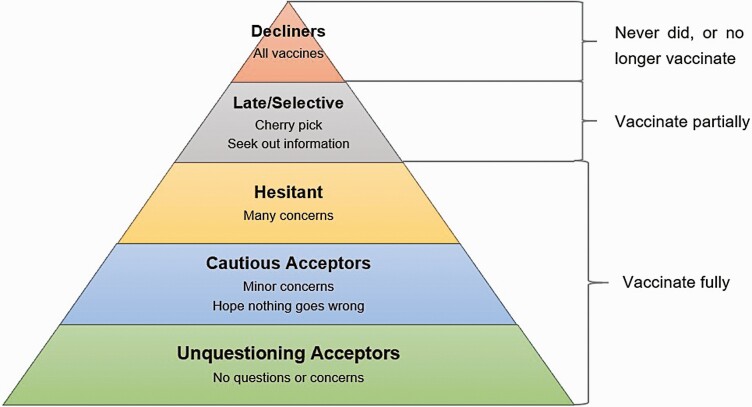
Vaccine acceptance spectrum. From: Leask, J. (2015, May 12). Improving communication about vaccination, adapted from Julie Leask’s online blog [5].

The WHO listed vaccine hesitancy as one of the top 10 threats to world health in 2019. The SAGE Working Group [2] has defined vaccine hesitancy as “the delay in acceptance or refusal of vaccination despite the availability of vaccination services.” Vaccine hesitancy has also been described as “the dynamic and challenging period of indecision around accepting a vaccination” [3]. As it is a complex and context-specific problem that varies across time and place and according to the vaccine and is also influenced by factors such as complacency, convenience, and confidence, vaccine hesitancy is not a clear-cut issue but represents a spectrum of beliefs and concerns, as shown in Fig. 1 [4, 5]. This paper defines vaccine hesitancy as: “The behavior and psychological concerns that cause people who are able to access vaccination to avoid doing so, either at all or in a timely manner” [2].

Although vaccine hesitancy is not a new phenomenon, the proliferation of antivaccination misinformation through social media has given it a new significance in the context of public health emergencies such as the coronavirus pandemic and hopes for the rapid development of a vaccine [6]. Misinformation can be a contributor to vaccine hesitancy. Although social media platforms help people communicate freely and share information, the rise of fringe opinions, misinformation, and disinformation also spread rapidly, damage public trust, and exacerbate vaccine hesitancy [6]. In particular, the growth of antivaccination groups has led to a sharp polarization of the debate and more people becoming skeptical of vaccination [7].

As well as being a potential source of the kind of (mis)information that causes hesitancy, social media offers two-way communication with target groups and individuals, increases the availability, accessibility, and appropriateness of the information, and provides a platform for low-cost and highly efficient policy advocacy opportunities [8]. It can provide an unprecedented opportunity to directly address public health issues and thus become a significant health promotion tool that is able to support behavior change [9]. Moreover, in the context of vaccine hesitancy, public health promoters have been encouraged to make greater use of social media, to be active rather than reactive regarding vaccination scares [10–12], and use network analysis and social computing methods to understand information spread through social media by antivaxxers and to support the development and implementation of social media interventions [13–15].

However, because social media is not a static property but a constantly growing and developing technology that could cause a broad and complicated set of behaviors, interactions, and exchanges involving numerous types of interconnected actors, precise definitions are somewhat elusive [16]. This review describes *social media* not only just as a medium of communication, but also as an online environment that enables individuals to socialize and a platform that features “interaction” as its main function [8]; we define *social media interventions* as “interventions that use social media as a tool to deliver intervention content with social media components aimed at influencing vaccine attitude or behavior.”

Vaccine hesitancy is a highly context-specific problem [17]. The extensive literature on the subject seeks to identify the determinants and barriers in people who are hesitant. Studies have found that these factors included contextual influences (such as social norms, religious beliefs, economic or political factors, trust in government and providers), individual and group influences (personal perception of the vaccine, the opinions of family and peers), and issues directly related to the vaccine (such as vaccine safety and ingredients) [2]. As a result, although many behavioral determinants and barriers to different vaccinations have been identified, few existing interventions have been explicitly designed to address vaccine hesitancy, and even fewer studies have quantified the effectiveness of such interventions in terms of increasing the knowledge and improving the attitude or behavior [3, 8, 18].

Many studies have shown that interventions informed by behavior change theories based on evidence-based principles are more effective than those that lack a theoretical framework [19–21]. In the existing literature pertaining to vaccine attitudes and intentions, behavior change theory is more often used to identify the behavioral determinants than to design and evaluate interventions [22]. Research actions informed by theory rather than by researchers’ intuition and experience have the potential to increase understanding of behavioral processes, enable the systematic design of evidence-based interventions, and help put behavioral science into practice. Theory-based interventions offer an opportunity to better understand the factors that drive vaccine hesitancy and to design interventions to address it. To the best of our knowledge, the literature has not yet been systematically examined to explore how behavior change theories have been implemented in interventions that seek to understand and address vaccine hesitancy.

This review sought to identify social media tools and behavior change theories used in vaccine hesitancy studies and assess their implementation and effectiveness in informing the systematic development of novel interventions for improving vaccination rates.

## METHODOLOGY

**Fig 2:**
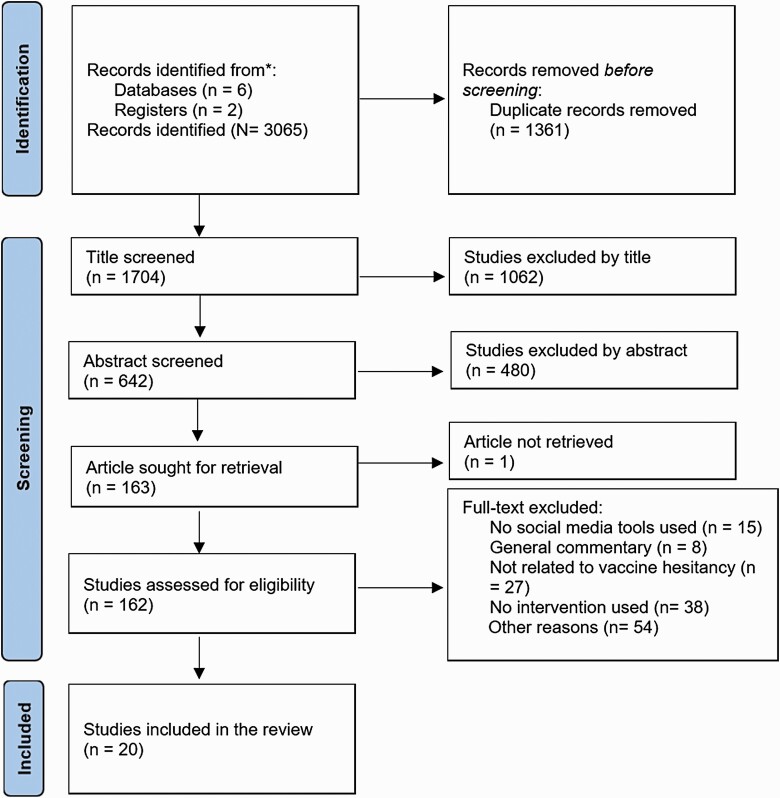
PRISMA diagram detailing the process of identifying and selecting the studies.

The PRISMA approach (Preferred Reporting Items for Systematic Reviews and Meta-Analyses Selection [23]) was used to search for articles from research library databases. Fig. 2 shows the selection process aligned with the PRISMA guideline. The publications were selected using predefined keywords and selection criteria as follows.

### Search strategy

We designed an organized structure of key terms to search literature in electronic databases (for search strategy and search keywords, see Appendix A). As each database works differently, the commands were adapted to obey different rules. Relevant literature published between January 1, 2011, and January 1, 2021, were collected by performing searches in PubMed, Scopus, ACM, Cochrane, ProQuest, and the Web of Science database.

### Eligible criteria

#### Inclusion criteria

1. Intervention and vaccination: vaccine hesitancy studies that use social media to deliver interventions to influence vaccine behavior (studies measuring people’s behavior toward vaccination) or increase vaccine confidence (studies measuring people’s change of knowledge, intention, or attitude toward vaccination).
2. If there was no explicit method shown in the title or abstract, full-text screening was carried out.
3. Only peer-reviewed articles published in English were included.

#### Exclusion criteria

Any article on the following subjects was filtered out:

1. Vaccine development processes for a particular disease; implementation of a general immunization program in a particular area that focuses on the whole vaccine allocation process, including governance, workforce management, vaccination sessions, and vaccine supply.
2. Theory-based interventions without using social media.
3. Interventions aiming to increase the utilization of immunization services or improve the availability of immunization services by healthcare providers.
4. Face-to-face or school-based educational methods without using social media intervention.
5. Behavior theory used for vaccine adjuvants, rabies, and school-based health promotion programs.
6. Commentary paper on social media and vaccine hesitancy without giving the intervention development process.

### Data extraction

All articles that accorded with the search strategy were collated, and duplicates were removed. Next, the authors reviewed the remaining titles and abstracts and excluded the articles that did not meet the inclusion criteria. The full text was evaluated if the title or abstract were inadequate for determining eligibility. The final included articles were divided into four categories based on the three types of social media intervention identified in the European Centre for Disease Prevention and Control (ECDC) social media intervention report [8] and the fourth category identified by the authors:

- Educational posts: interventions using social media as a communication channel to post or promote educational information to the public.
- Dialogue-based: interventions engaging the public in online discussion groups.
- Interactive website: interventions utilizing interactive websites with integrated social media components to deliver information and engage users.
- Personal reminder: interventions providing personal reminder information using social media channels.

The authors extracted the key points from the included studies, including study details (study area; targeted groups; sample size; vaccine; intervention type; social media type and theory), study aims, intervention details, outcome, and results.

### Risk of bias assessment

The risk of bias for each included study was assessed by the authors using the adapted MetaQAT form (the Public Health Ontario Meta-tool for Quality Appraisal for Public Health Evidence [24]) to guide the critical appraisal process. As shown in Appendix B, three domains accounted for the overall judgment: relevancy, reliability, and validity. The included studies were scored medium and high in total score (13 articles were rated between 7 and 9 [25–37], seven studies were between 4 and 6 [38–44]). Then the overall score for each domain was counted.

The validity domain presented the lowest score due to the potential methodological bias. Among these, the issue of small sample size was the most frequent issue which has been found in six studies [25, 26, 28, 36, 38, 44], followed by the lack of generalization in six studies [25, 28, 33, 37, 38, 40], and response bias caused by self-reported measures in three studies [26, 35, 39]. Three studies also reported the inability to measure the outcome due to the study design that leads to the difficulty in distinguishing the impact factors [33, 35, 40]. Besides, the study also reported the potential social desirability bias and in-group bias [31] and the potential weakness on outcomes biased by baseline vaccine hesitancy [38, 41]. Despite the potential biases, the studies elucidated that these methodological issues were minimized by using various strategies.

## RESULTS

The literature search yielded 3,065 publications from all data sources. Twenty articles met the inclusion criteria. Of these, eight studies used social media to deliver the interventions without using behavior change theory, and twelve studies were informed by behavior change theories. The study characteristics in each group were shown in Appendix C, which displays the study details, including aims, study design, a brief description of the intervention, and results. In this section, we will first present the general description of the included studies, then describe the studies from the perspectives of the social media use and behavior change theory, and present the effectiveness and evaluation of the included studies qualitatively.

**Fig 3:**
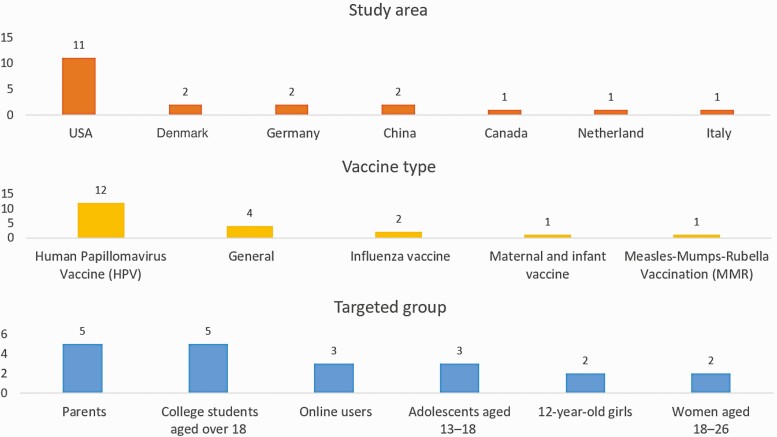
Study overview of the included articles.

The study overview of the included studies is displayed in Fig. 3. Most of the studies were conducted in the United States (*n* = 11); other in Denmark (*n* = 2), Germany (*n* = 2), China (*n* = 2), Canada (*n* = 1), Netherland (*n* = 1), and Italy (*n* = 1). Twelve interventions addressed HPV vaccination, two on influenza vaccination, one focused on maternal and infant vaccination, and one on Measles-Mumps-Rubella vaccination (MMRs). The remaining four addressed vaccinations in general. In terms of the targeted groups, most of the studies focused on the people aged 12–40, including parents (*n* = 5), college students (*n* = 5), adolescents aged 12–18 (*n* = 3), girls aged 12 (*n* = 2), and women aged 18–26 (*n* = 2); the other three studies focused on general online users without giving specific age.

### Use of social media components

Thirteen interventions used a single type of social media platform (Facebook (*n* = 9) [28, 30, 32–35, 38, 40, 42]; YouTube (*n* = 1) [36]; Twitter (*n* = 1) [25]; WhatsApp (*n* = 1) [31]); three used general online forums [26, 27, 29]; and one used a mixture of social media tools (Twitter, Facebook, and Vine [44]); the other three used websites with a built-in interactive component [37, 39, 41]. In terms of the type of intervention, the most common was educational posts (11 articles), followed by dialogue-based (5 articles), interactive website (3 article), and personal reminders (1 article).

#### Educational posts

Social media campaigns that post educational messages to the general public were the most common method [25, 30, 32, 33, 35, 36, 38, 40, 42–44]. People (mostly women) received daily educational messages, including personal stories, medical opinions, facts, and related events, produced by public health agencies through Facebook or YouTube over 1 month to 1 year.

These studies highlighted the effect of different types of contents and ways of posting: Loft et al. investigated the impact of different types of contents on engaging and reaching the audience and concluded that posts containing personal stories performed better than that containing factual information [32]; La Torre et al. found that events were the most popular type of news compared to press releases and publications and Friday is the day when the contents attracted more watches and likes [42]; Robichaud et al. compared the effect of evidence-based and anecdotal video and concluded there is no significant difference in participants’ attitude and behavior after watching the videos [36]; Lee and Cho investigated whether different message framing had a various impact on the public’s behavior intention to get vaccinated and found participants who received the loss-framed message on Facebook mostly exhibited a higher level of behavioral intention than those in the gain-framing condition [30].

The interventions using educational posts were always combined with other types of interventions. For example, Gerend et al. combined social media posts with posters and yard signs around the campus to promote the HPV vaccine, and it observed a 75% increase in vaccine uptake among the targeted group [40]. Besides, it could also be integrated with other modes of social media. Pedersen et al. carried out a social media campaign to engage Danish girls in communication regarding the HPV vaccine, and the strategy was proved useful [35]. Mohanty et al. used Facebook to reach adolescents for promoting the HPV vaccine while sending reminder letters to the participants [33].

#### Dialogue-based interventions

Five studies used the dialogue-based social media interventions, three of which were carried out through online forums [26, 27, 29], and the others were conducted in social media platforms (Facebook (*n* = 1) [28]; WhatsApp (*n* = 1) [31]). Participants took part in forums in which they discussed different subjects according to their area of hesitancy. Kimmerle et al. examined the difference of impacts between using factual information versus using personal experience on subsequent communication [27]. The results demonstrated that both kinds of information had a similar effect in engaging users, but the responses to personal experience were significantly more emotional. Hasse et al. attempted to assess the effect of the credibility of the post source on the participant’s risk perception toward vaccines, but the result shows the discussions on vaccine adverse events decreased the vaccine intention and increased risk in both neutral and antivaccination online health forums [26].

It can also be combined with other social media modes. For example, Liao et al. promoted the childhood vaccine through discussion groups among parents while sending weekly vaccination reminders, and they identified the most common discussion topics including vaccination decisions, vaccination clinic and cost, vaccine safety and side effects, and vaccine effectiveness, and also found the combined intervention effectively promote mothers’ self-efficacy in making vaccine decision [31]. Besides, the dialogue-based interventions can assist the vaccination promotion programs in school settings. Lai et al. designed an intervention for educating students about the HPV vaccination in lectures with two intervention groups in China and concluded the Facebook-assisted discussion method was more effective than the in-person discussion [28].

#### Interactive websites

Interactive websites provided evidence-based information in an interactive environment through social media. By incorporating social media components such as Instagram, Facebook, Twitter, YouTube, the website could be used to share and interact with other users and receive responses from doctors and other experts. Notably, all three studies were contributed by one research team with a different focus: Glanz et al. developed the interactive social media website that aims to increase childhood immunization by tailoring the content, design, and information architecture toward parents who are seeking to make informed decisions regarding vaccination for their children [37], and they also carried out a series of studies to compare the effectiveness of website without social media, website with social media and usual care on improving parents’ vaccine-related attitudes and found pregnant women exposed to the social media were more likely to vaccinate their infants on time than participants receiving usual care [39, 41].

#### Personal reminders

The personal reminders intervention uses a software system to record the data and send the automated reminders through social media to targeted participants. In some cases, personal reminders were always combined with other intervention content and methods to encourage participants to change their behavior [31, 33]. Only one study used personal reminders solely to deliver the intervention content in this review, which developed a reminder system by using a mixture of reminder channels—text message, e-mail, phone call, Facebook message, and standard mail to remind the participants to get the HPV vaccine; however, the result shows the intervention did not increase HPV vaccine completion [34].

### Behavior change theory

**Fig 4:**
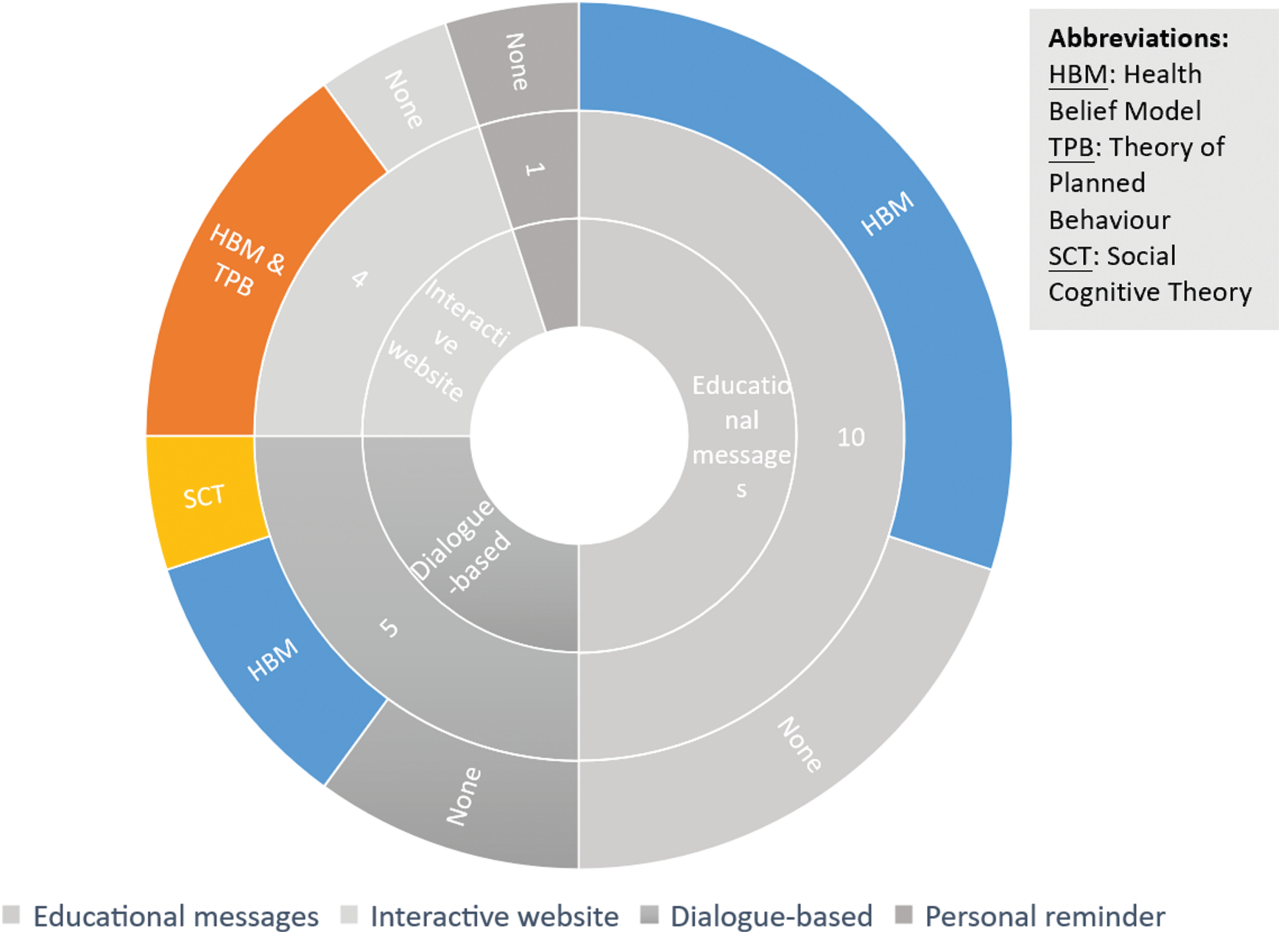
Type of social media intervention and the use of behavior change theories.

Nine studies reported using behavior change theories to inform the design of social media interventions. The Health Belief Model, Theory of Planned Behavior, and Social Cognitive Theory (SCT) were employed in these studies. Figure 4 presents the use of behavior change theory in each social media mode of delivery.

#### Health Belief Model

Eight studies used the Health Belief Model (HBM) alone, and the other three combined HBM with the Theory of Planned Behavior. As shown in Fig. 4, six single-theory studies are delivered through educational messages, while two were through dialogue-based social media interventions. The HBM posits four principal drivers of health behaviors: people’s knowledge and perception of the severity of disease and their susceptibility to it; people’s perception of the barriers to and benefits of being vaccinated; self-efficacy, that is, their belief in their ability to be vaccinated; and the cues to action. The model also assumes that the intention to enact a certain behavior will be followed through [25, 45], so it can be used as a framework to evaluate the effectiveness of the intervention on behavior change. Most of the studies that used this model were based on the hypothesis that people with higher perceived susceptibility, higher perceived benefits, and higher self-efficacy can be defined as lower-barrier groups that are more likely to engage in behaviors that promote health [25, 46, 47].

Of the eight studies, all reported that the HBM was employed as a guiding theory for intervention development: three were based on all components identified by the HBM [33, 43, 44], four excluded the consideration of cues to action [25, 28, 30, 38], and one study only focused on cues to action [29]. Only four applied the same theoretical framework to evaluate the effectiveness of interventions [25, 28, 29, 44]. In terms of way of incorporation, it was slightly different with different social media modes. For the educational posts, the campaign messages or contents were simply constructed to align with the HBM components [25, 38, 44], whereas for dialogue-based interventions, the HBM was used as a rationale to design the whole intervention process [28, 29]. For example, Allen et al. provided a series of sample tweets for each HBM component, and the messages were sent to the participants to address the targeted barriers [25]; but in Langley et al.’s study, they designed the dialogue-based interventions to prompt external cues to encourage the vaccine behavior [29].

#### Theory of planned behavior

As mentioned, three studies combined the Theory of Planned Behavior (TPB) with HBM. According to the TPB, the behavior could be directly influenced by intentions, which are determined by attitudes (a function of behavioral beliefs), subjective norms, and perceived behavioral control (a function of control belief) [48]. All the three studies were the intervention delivered through interactive websites by one research team in the same way, that is, the multitheory model was first used in the design of the factual vaccine content to present on the website, and then implemented as indicators in the measurement of effectiveness for assessing the attitude toward perceived benefits and risks of vaccination and self-efficacy regarding vaccine decision-making [37, 39, 41].

#### Social cognitive theory

SCT considers the six elements in addressing behavior change of individuals, which includes reciprocal determinism (the interaction of individual, environment, and behavior), behavior capability (perceived facilitators and barriers to action), vicarious learning (the source of information cognitive and behavioral development), reinforcements, expectations, and self-efficacy [49]. Compared to the HBM and TPB, SCT focuses more on the social motivation for achieving the behavior change. For example, in Liao et al.’s study, the intervention was developed to promote mothers’ self-efficacy through group discussion for taking their children to get the influenza vaccination, and the self-efficacy was served as the main indicator for design and measured effectiveness [31].

### Effectiveness and evaluation

This section comprises the effects of the interventions on improvement in knowledge, intention, behavior, or other outcomes. Due to the heterogeneity of the studies and a lack of suitable data for measuring effectiveness, we were unable to compare the effectiveness quantitatively. Instead, we collected the primary outcomes of the included studies to present the effectiveness qualitatively.

As shown in Table 1, the evaluation approach varies depending on the measurements. Theory-based Likert-type scales are the most used to evaluate knowledge, attitude, and intentions. For example, Lai et al. developed a 20-item self-report scale with a five-point Likert-type rating to assess the attitude and intention of HPV vaccination behavior post the intervention [28], which is similar to the methods used in other studies [25, 30, 36, 41, 44]. To evaluate the vaccine uptake rate, six studies used the medical records [33, 34, 37, 40, 41, 43], whereas the other three collected the data through self-reported surveys [31, 38, 44].

**Table 1:** Outcomes and the evaluation approaches of the included studies

Reference	Social Media	Theory	Outcome Type	Outcome Details	Evaluation Approach
Allen et al. [25]	Educational message	Health Belief Model	Knowledge	No statistically significant change (Pre 56% vs Post 57%, *p* = .858)	Survey by phone/online
			Intention	No statistically significant change in next 6 months (*p* = 1.000) or 12 months (*p* = .617)	Survey by phone/online
Brandt et al. [38]	Dialogue-based	Health Belief Model	Knowledge	Knowledge of HPV and HPV vaccination significantly improved among participants in the HPV vaccination awareness intervention (correct answer rate: 92.6%–100%, *p* < .05) relative to those in the control condition (correct answer rate: 65.5%–82.8%) among several questions.	Online questionnaires for all self-reported measures
			Intention	No significant differences were found between intervention vs. control groups: intension to get the vaccine in 6 months rate: 8% increased vs. 3% increased (post)	Online questionnaires for all self-reported measures
			Behavior	No significant differences were found between intervention vs. control groups; fully vaccinated rate: 12% increased vs. 4% increased (post)	Online questionnaires for all self-reported measures
Daley et al. [39]	Interactive website	Theory of Planned Behavior and Health Belief Model	Intention	Significant improvements in attitudes regarding vaccination benefits among social media intervention (VSM) (mean change 0.23 on a five-point scale, 95% CI = 0.05, 0.40) and information only intervention (VI) (mean change 0.22, 95% CI = 0.04, 0.40) vs usual care (VC);Significant reductions in parental concerns about vaccination risks (VSM mean change –0.37, 95% CI = –0.60, –0.14, VI mean change –0.31, 95% CI = –0.55, –0.07).	Online survey using a secure platform (SurveyGizmo)
Gerend et al. [40]	Educational message	None	Behavior	75% increase in HPV vaccine doses administered to students of all ages in 2018 vs. 2019 spring semester (290 vs. 509 doses), respectively; 18–26-year-olds (77% increase, 273 vs. 455 doses)	Electronic health record
Glanz et al. [41]	Interactive website	Theory of Planned Behavior and Health Belief Model	Behavior	Although none of the study arm (VSM/VI/VC, see Daley et al.) comparisons were statistically significant, infants in the VSM and VI arms were two times more likely to have received MMR than infants in the usual care (UC) arm.	Data were extracted from the electronic health record
Haase et al. [26]	Dialogue-based	None	Intention	A higher number of narratives reporting vaccine adverse events decreased intentions to get vaccinated (*p* < .01); In addition, when the narratives originated from a neutral online health forum, as compared to an anti-vaccination website, intention decreased (*p* < .01).	Online survey
Kimmerle et al. [27]	Dialogue-based	None	Other	No evidence that personal experiences evoked more responses (mean 3.79, SD 3.91) from other members of the Internet forums than fact-oriented contributions (mean 2.14, SD 2.93, *t*26 = 0.126, *p* = .219). But personal experiences elicited emotional replies (mean 3.17, SD 1.29) from other users to a greater extent than fact-oriented contributions (mean 2.13, SD 1.29, *t*81 = 3.659, *p* < .001)	User records
La Torre et al. [42]	Educational message	None	Other	Engagement by three indicators: friend membership (743), numbers of “I like” (247), amount of “share” of contents for the type of news (62), and day of the week (highest; mean: 2.20; min 1, max 8)	User records
Lai et al.[28]	Dialogue-based	Health Belief Model	Knowledge	Improvement of knowledge score was (pre vs. post) 4.204 and 4.496 points	Hard copy ofquestionnaires with a 20-item self-report scale and a 5-point Likert-type rating
			Intention	Improvement of attitude score was (pre vs. post) 4.496 points; The Facebook group’s intention improvement was 2.310 times greater than the control group’s improvement (out-of-pocket expenses) and 2.368 times greater (free).	Hard copy ofquestionnaires with a 20-item self-report scale and a 5-point Likert-type rating
Langley et al. [29]	Dialogue-based	Health Belief Model	Intention	Not available	Questionnaire
Lee and Cho[30]	Educational message	Health Belief Model	Intention	Content: a significant interaction effect between message framing and media channel, *F* (1, 141) = 4.60, *p* < .05: loss-framed message on Facebook (*M* = 4.90, *SD* = 1.33) higher than gain-framing condition (*M* = 4.03, *SD* = 1.37), *p* < .05.Channel: no statistically significant medium effect between newspaper (*M* = 4.49, *SD* = 1.39) and Facebook (*M* = 4.47, *SD* = 1.41), *t* (140) = .08, *p* = .94.	Survey through a 7-point Likert-type scale questionnaire
Liao et al. [31]	Dialogue-based	Social Cognitive Theory	Intention	The intervention significantly promoted mothers’ self-efficacy for taking children for the seasonal flu vaccine (SNI–TP: odds ratio [OR] 2.69 [1.07–6.79]; SNI+TP: OR 2.50 [1.13–5.55]). SNI (social networking intervention); TP (time pressure)	Interview and survey
			Behavior	No significant improvement in children’s seasonal flu vaccine uptake	Interview and survey
Loft et al. [32]	Educational message	None	Other	Reach number: 3,476,023 individual FB profiles by 84 posts; personal stories generated higher engagement rates and more positive dialogues than factual posts.	User records
Mohanty et al. [33]	Educational message	Health Belief Model	Behavior	Overall, 176 doses of the HPV vaccine were administered to 152 unique adolescents.	Medical records (Immunization Information System)
Ortiz et al. [43]	Educational message	Health Belief Model	Knowledge	Pretest to posttest difference between the groups for knowledge gain, *F* (3, 103) = 2.76, *p* < .05;Participants who discussed with others had significantly higher knowledge scores at both pretest (*M* = 4.51, *SD* = 2.29) and posttest (*M* = 5.33, *SD* = 2.44) than those who did not (pretest: *M* = 3.12, *SD* = 2.23; posttest: *M* = 4.27, *SD* = 2.44), *F*(1, 80) = 58.84, *p* < .05	Online survey questionnaires with eight relevant questions assessment
			Behavior	No significant increase for vaccination rates, *F* (3, 103) = 1.10, *p* = .35.	Medical records (Immunization Registry)
Patel et al. [34]	Personal reminder	None	Behavior	The reminder system did not increase completion rates, which overall were low at 17.2% in the intervention group and 18.9% in the control group (*p* = .881).	Medical records
Pedersen et al. [35]	Educational message	None	Other	Reach number: 8,020,000 people with an average of 127 comments per post. The average Engagement Rate (ER) from May 2017 to halfway through 2018 was 6.07%, and the Click Through Rate (CTR) was 2.09%. Personal stories were the most effective in creating positive dialogue. One year later, the number of positive comments increased from less than 50% to approximately 75%.	User records
Robichaud et al. [36]	Educational message	None	Intention	No significant difference in pre to post attitudes toward influenza immunization nor were there any differences when comparing the two different vaccine-critical videos	Likert-style survey questions
Shoup et al. [37]	Interactive website	Theory of Planned Behavior and Health Belief Model	Knowledge	71%–100% of the parents (*n* = 443) were able to complete specific tasks, and between 14% and 100% of the tasks were completed without error. Approximately 50% of parents said they would discuss their experiences and vaccine concerns using the web-based intervention tool	User records
			Behavior	Not available	Screening medical records in next two years
Sundstrom et al., 2018[44]	Educational message	Health Belief Model	Knowledge	Unclear	An anonymous web-based survey
			Intention	High levels of intention to be vaccinated	An anonymous web-based survey
			Behavior	60% (n=237) of Participants indicated that they received the HPV vaccine due to the intervention	An anonymous web-based survey

Fifteen studies reported the effectiveness on knowledge gain, intention, or behavior change; one study stated in progress without provided any result [29], and other four studies used other measurements instead, such as engagement rate and click-through rate [32, 35, 42] and the number of replies [27]. Among the fifteen studies, eleven of them were theory-based interventions, and only four nontheory-based studies reported the effects on intention or behavior change [26, 34, 36, 40], two of which are even no significant change [34, 36]. One nontheory-based study reported a 75% increase in HPV vaccine uptake in a school design [40]. Among the other six theory-based studies that reported behavior change outcomes, one indicated no statistically significant difference between intervention and control groups, although the higher completion rate was shown in the intervention group (12% vs. 4%) [38]; two also reported no significant increase for vaccination uptake [31, 43]; and the other three studies reported positive effects. One reported 60% of participants received the HPV vaccine due to the intervention [44], another reported a significant difference between control groups that participants in the social media intervention group were two times more likely to receive the MMR vaccine than those in usual care and internet intervention groups [41], and the third reported the number of doses and participants administered for the HPV vaccine (176 doses and 152 unique adolescents) after the intervention [33].

In terms of the mode of delivery, four theory-based education posts reported significant results: one found higher knowledge gain between before and after the intervention, but no significant increase in vaccination rate [43]; one reported the unframing message promoting through Facebook significantly increased the vaccine intention [30]; the other two reported higher vaccination uptake rate due to the intervention [33, 44]. Another nontheory-based educational post also reported a higher vaccination rate with a 75% increase among the participants after the intervention [40]. The theory-based interactive website had significantly improved the vaccine knowledge [37], attitudes and intention to vaccinate [39], and vaccine uptake [41]. Three dialogue-based interventions were reported to be effective in improving people’s intention to be vaccinated [28, 31], one of which is nontheory-based intervention [26]. It also performed well in increasing the vaccine knowledge and awareness [28, 38], although two studies reported no significant improvement was found in the vaccine uptake [31, 38].

## DISCUSSION

Here, we highlight the key findings—the effectiveness, strengths, and limitation of using each social media component and behavior change theories in the interventions to address vaccine hesitancy, then summarize the opportunities and challenges that face future attempts to integrate behavior change theory and social media interventions as a means of addressing the problem of vaccine hesitancy, and eventually identify the benefits and limitations of this research.

### Social media use

The included articles were divided into four categories according to the main intervention type, although some had multiple components. Each mode has its advantages and disadvantages.

The chief merits of educational posts are the low cost and the potential for wide coverage [40]; an apparent weakness, however, is the use of nontailored information. Furthermore, with no control group among so many participants, the effects of other factors cannot be ruled out. It is difficult, therefore, to measure the effectiveness of this type of intervention [35]. The alternative would be to narrow down the target group and provide tailored messages for specific populations, such as college students or parents, and offer guidance on how and where to access the vaccination [40]. Such tailoring is always more effective, but the cost is greater.

For the dialogue-based interventions, participants could post their opinions and concerns about the vaccine or disease and freely communicate with other participants about their experiences; therefore, it is much easier to evaluate the effectiveness by collecting the data using repeated surveys than the educational posts. The researchers were able to observe the influence of initial posts and follow-up discussions on changes in participants’ attitudes [27–29, 31, 38]. Besides, the results show that information from discussion groups can effectively improve vaccine confidence by enhancing the related knowledge and dispelling the doubts, but this alone may not be enough to solve the vaccination concerns and enable people to make positive vaccination decisions [28, 31, 38].

Similarly, the questionnaires could be sent to participants to assess their trust in the social media tools and the health information received for the interactive websites. Besides, its multicomponent nature facilitates it to be more functional—participants can receive the educational information from the website and discuss their questions and concerns with other users and receive responses from doctors and other experts. However, its development, engagement, and management cost is much higher than the first two. It is worth noting that the study found the contents using storytelling approaches and answers to questions posed by readers on the website show the highest number of reads and engagement [32].

A personal reminder has the advantage of being easy to measure the effectiveness. Apart from using a self-reported survey, personal medical records were also used to evaluate the effectiveness of the interventions; researchers could observe how many participants delayed or refused vaccination for personal or nonmedical reasons. However, it alone presents the lowest effectiveness compare to other intervention types, and consequently, it should be combined with other intervention components [34].

In terms of effectiveness, we found that the strength of different modes lies in different targeted outcomes. The educational post performed well in improving knowledge, while dialogue-based intervention is better in increasing the intention or change the attitude, but may not be enough on their own to trigger behavior change. Thus, the most effective interventions employed multiple strategies. In this review, we found that the most significant increases in vaccine uptake on the first dose (increases of up to 75%) were observed with interventions that used educational messages with posters and signs [40], particularly those that embedded information about to recommend HPV vaccination to others. Such increases could nonetheless have other causes that are difficult to distinguish. In general, we found the interventions that reported the significant increase in vaccine uptake were those that: (1) targeted specific populations; (2) included tailored content; and (3) used behavior change theory in their design.

Summarizing the benefits and limitations of social media interventions is problematic because the studies were carried out in various countries and cultures and are therefore highly context-specific. In general, evidence-based social media interventions have the potential to significantly improve the knowledge, intention, and behavior about vaccines and clear the behavior barriers, especially when compared to other modes such as mass media [30] and the general internet [37, 39, 41]. Additionally, there are undeniable benefits to using social media—fast access to a wide audience, low cost—so its future is promising, but more evidence-based strategies and studies are needed.

Three main limitations to these studies can be identified. The first is the difficulty in clarifying the relationship between the effects on the behavior of the social media intervention itself and the effects of other factors [28, 33, 34]. Most studies measure knowledge and attitudes, risk perception and concerns, and the intention to be vaccinated. Tracking vaccination uptake by accessing participants’ medical records could help address this shortcoming, but there are apparent issues here regarding confidentiality. Second, although studies suggest that social media interventions could be effective for people who are already open to vaccination or who already recognize the role of social media in the field of health and wellbeing, it is well understood that social media platforms are vulnerable to exploitation and have become a forum for antivaccine discussions [3]. Third, social media interventions have the potential to exacerbate disparities in healthcare that result from differences between social groups regarding access to the internet and to smartphones [25, 35, 40]; people without access to social media can become marginalized or completely excluded.

### Behavior change theory

Across the studies included in this paper, we identified the behavior change theories used in interventions to influence vaccine uptake, which are the Health Belief Model and the Theory of Planned Behavior, and SCT. However, since only nine studies were identified in this review, additional research is needed to provide more robust evidence for the utility of these theories. Besides, considering only a small number of theories are being implemented in the design of social media interventions, there is scope for expanding the use of theory to provide a greater understanding of vaccine hesitancy and inform the design of interventions aimed at addressing it and improving vaccine uptake.

A clear difference was identified between the studies with and without theory—theory-based studies are more measurable and replicable. The unique role of theoretical methods is their function on bridging the gap between knowledge and behavior, which could expand the function of intervention from knowledge, attitude to intention and behavior. In terms of the application of behavioral theories, we found that in addition to directly guiding the interventions, many studies only used the theories to design parts of the intervention content, such as videos, articles, and stories, or to measure the effects of interventions [33, 37–39]. Notably, only three studies have been consistent in their design and evaluation, using the same framework to measure effectiveness [25, 28, 44].

Through the qualitative evaluation on the effectiveness, we found that the Health Belief Model has great potential in improving vaccine knowledge, intention and uptake, although HBM-based studies also identified no significant improvement in knowledge, intention, or vaccination rate [25, 38, 43]. Among the HBM components, we found cues to action is the most underdeveloped and least researched element, which is in accordance with the previous studies [50], especially the external cues. However, since vaccination is a social contract wherein getting vaccinated is the morally right behavior [51], which needs endorsement from the social environment. This might be a possible explanation for the low effectiveness of the three low effective studies.

Meanwhile, it highlights the need for a multitheory-based framework that can take all determinants into account. Considering vaccine hesitancy is a behavior in a complex network of related behaviors, changing that one behavior is likely to influence the other components of the system and reconfigure the whole network. In this case, a systematic framework considering a wide range of options would aid the development and implementation of any intervention [52]. Furthermore, although behavior change models such as HBM and TPB have been used to inform the interventions and may have some effect, they may suit only one specific circumstance and have limited value. A framework based on multiple theories, such as Behavior Change Wheel [53], could facilitate the evaluation process by providing tools to diagnose possible loopholes in the development of the intervention and so improve the design and achieve the desired goal.

### Recommendations for future interventions

Our review demonstrates that behavioral theory-based social media interventions have the potential to improve vaccine knowledge and confidence, whereas there is insufficient evidence for its function on the effectiveness of increasing vaccine intention or uptake. Most of the findings from the reviewed studies show that these approaches were effective in improving knowledge, attitudes, or belief [30, 31, 33, 37–39, 41, 43, 44, 54], but only a few addressed vaccine concerns sufficiently to achieve a positive vaccination decision [33, 40, 41, 44]. This result may be explained by the fact that most social media interventions tend only to inform and educate participants; taking people on a journey from social media exposure to changes in vaccine behavior is proving a challenge. Based on our findings, we have several recommendations for future studies.

First, future work is required to focus on the behavior change process by adopting the appropriate mode according to the context and targeted outcomes. This review has summarized the strengths and limitations of the different social media components, which could facilitate the better incorporation of behavior theory and social media mode in future interventions.

Second, the targeted outcome should be shifted from knowledge improvement to intention and behavior change. Studies have confirmed that people can remain highly suspicious of vaccines despite the facts and the evidence and that knowledge alone is not enough to initiate behavioral change [55]. Using behavior change theories to better understand multiple factors, such as beliefs, motivations, and socio-cultural influences, and integrating these theories with social media interventions, is seen as one way to move people from merely receiving information to changing their behavior [18].

Third, this review highlights the need to use a multitheory-based framework to inform and evaluate the interventions. It is not only because the multi-theory-based framework has more potential to consider more determinants and trigger the behavior change in the whole system, but also because it can better facilitate the measurement and comparison between different interventions. In this review, we found that although some studies have already used behavior change theories to guide and inform the development of interventions, the effectiveness of those interventions, especially those that use social media campaigns without reporting effectiveness on behavior outcomes, remains difficult to gauge. Furthermore, especially for studies based on behavior change theories, inconsistencies in terminology make comparisons yet more problematic. Interventions based on a single study provide insufficient data for assessing how successful the intervention would be among a broader population or with a different vaccine.

There are undoubtedly some lessons to be learned from the existing studies on implementing theory-based social media interventions. First, social media alone is not an intervention but a channel for facilitating engagement between people who want to interact with others [37]. Therefore, strategies need to be adopted to ensure the accuracy and coherence of the information being provided, and experts in the relevant fields need to be in place to moderate group discussions [37, 39, 41]. Second, positive behavioral change is more likely to happen if there is a strong trust between the researchers and the target group [37]. Such trust can be fostered by ensuring transparency in all communications, acknowledging participants’ concerns, and responding promptly to their questions with tailored information.

### Limitations of this systematic review

To the best of our knowledge, this is the first broad-based and systematic review of social media interventions that aim to increase vaccine confidence with respect to all vaccine types and populations. The evidence appears to support and strengthen the feasibility of using theory-based social media interventions to address vaccine hesitancy.

Nonetheless, some limitations in the review process and research design have been identified. First, our review covered all types of vaccines worldwide, but vaccine hesitancy is a highly contextualized problem. The utility of the results is therefore limited. The studies included are at different stages, so the results on effectiveness need to be interpreted with caution. Second, some methodological issues should be mentioned. The articles collected were from only six databases (PubMed, Scopus, ACM, Cochrane, ProQuest, and Web of Science), they were all published between January 1, 2011, and January 1, 2021 in the English language, and no consideration was given to grey literature. The variation in research design, methods, and outcomes further reduce the feasibility of making comparisons between the various findings. The results of the studies are difficult to quantify, but the number of publications can at least be used to reflect the amount of research being carried out in the different disciplines. Finally, although the findings of some of the broader studies could be applicable to a wider population, the overall heterogeneity of the research led to a limited generalization of the conclusions.

## CONCLUSION

The purpose of this review was to identify the breadth and effectiveness of behavior change theories and social media tools used in interventions aimed at addressing vaccine hesitancy. The review analyzed 20 studies from 3065 search results according to our search strategy. To the best of our knowledge, this is the first review to examine the use of behavior change theories and social media as tools for delivering vaccination interventions. The review has found that generally theory-based interventions were defined with more rigor allowing for quantifying and comparing outcomes. The most common theory and social media tools used in the previous interventions were Health Belief Model and posting educational messages. Educational posts were proved to be effective in increasing knowledge but less helpful in triggering behavior change. Dialogue-based social media intervention performed well in improving people’s intention to vaccinate. It also highlights the importance of using a multitheory framework and of tailoring social media interventions to specific circumstances. However, caution must be applied with a small number of studies included and the heterogeneity between the studies. Despite the limitations, the initial results appear to support the view that interventions informed by theory and delivered via social media platforms offer a significant opportunity and a valuable avenue to address vaccine hesitancy. This review could provide guidance for the future development and proliferation of such interventions.

## Supplementary Material

ibab148_suppl_Supplementary_File_A

## Appendix A Keywords

**Table:**

Keywords	Results
TITLE-ABS-KEY: (twitter OR facebook OR “online social network” OR “social media” OR sns OR Instagram*) AND (*vaccin* OR immuniz* OR immunis* *OR jab* OR Shot*) AND (*intervention OR strateg*) OR TI(vaccin* OR immuniz* OR immunis* OR shot? OR jab?) AND AB,TI,IF(((behavio?r* OR psychology OR psychological* OR attitude?) AND (chang* OR alter* OR modif* OR diagnosis OR determinant? OR framework? OR intervention* OR theor* OR model?)) OR (theor* adj2 (“reasoned action” OR “normative conduct” OR “social cognitive” OR “self efficacy” OR “habit goal” OR “health belief”))) AND AB,TI,IF(Intervention OR strateg*) NOT AB,TI,IF(clinic* OR survilliance OR monitor* OR recruit* OR animal OR vateri*)	Proquest:654 Scopus: 987 Web of Science:626 PubMed:262 Cochrane: 487 ACM: 49

## Appendix B Risk of bias assessment (meta QAT)

**Table:**

Reference	Assessment of Relevancy	Assessment of Reliability			Assessment of Validity				Overall comments on study	Total
Meta QAT Question	Does the study address a topic(s) relevant to the issue under investigation?	Is the study presented clearly?	Does the methodology describe the population studied, the intervention given, and the outcomes?	Are ethics procedures described?	Is the study methodology appropriate for the scope of research?	Is the research methodology free from bias?	Are the authors’ conclusions explicit and transparent?	Can I be confident about the findings?	Can the results be applied within the scope of public health?	
Allen et al. [25]	1	1	1	1	1	1	1	Unclear	0	7
Brandt et al. [38]	1	1	1	0	1	0	1	1	0	6
Daley et al. [39]	1	1	1	0	1	0	1	1	0	6
Gerend et al. [40]	1	1	1	0	1	Unclear	0	1	0	5
Glanz et al. [41]	1	1	1	0	1	0	1	1	0	6
Haase et al. [26]	1	1	1	1	1	0	1	1	1	8
Kimmerle et al. [27]	1	1	1	1	1	Unclear	1	1	1	8
La Torre et al. [42]	1	1	1	0	1	Unclear	0	Unclear	1	5
Lai et al. [28]	1	1	1	1	1	0	1	1	0	7
Langley et al. [29]	1	1	1	Unclear	1	Unclear	1	1	1	7
Lee and Cho [30]	1	1	1	1	1	Unclear	1	1	Unclear	7
Liao et al. [31]	1	1	1	1	1	0	1	1	1	8
Loft et al. [32]	1	1	1	0	1	1	1	1	0	7
Mohanty et al. [33]	1	1	1	0	1	1	1	1	0	7
Ortiz et al. [43]	1	1	1	0	1	Unclear	1	Unclear	Unclear	5
Patel et al.[34]	1	1	1	1	1	0	1	1	Unclear	7
Pedersen et al. [35]	1	1	1	0	1	1	1	1	Unclear	7
Robichaud et al. [36]	1	1	1	1	1	1	1	1	0	8
Shoup et al. [37]	1	1	1	1	1	1	1	1	1	9
Sundstrom et al. [44]	1	1	1	1	1	0	Unclear	1	0	6
Total	20	20	20	10	20	6	17	17	6	

## Appendix C Study characteristics

**Table:**

Reference	Study Details	Aims of Study	Outcomes and Results	Intervention Details
Educational messages				
Loft et al. [38]	Country: Demark Sample size: 84 posts Targeted group: 12-year-old girls Vaccine: HPV Intervention type: Facebook (FB) posts Social media type: Facebook Theory: None	Assess how different Facebook (FB) posts resonate with parents hesitant about HPV vaccination and how to engage parents in positive dialogues on FB.	The study findings confirmed that the campaign has been successful in engaging the FB users. The descriptive analyses show higher engagement, higher reach and more positive sentiments for the posts that contained personal stories compared to those containing factual information. Parents engaged with different types of content; however, the engagement was highest when the content was personal and less factual, even if the factual information related to the safety of the vaccine. This is not in accordance with the opinions obtained from focus groups where the participants requested more in-depth information, including the safety of the vaccine.	In order to assess the performance of different types of content, They categorized the posts on the FB page into six subgroups: 1. Personal stories: Stories from people’s everyday lives related to HPV and HPV vaccination e.g. women telling their story of having cervical cancer 2. Medical opinions: Stories from health professionals about their work experiences with HPV related diseases and facts about HPV vaccination 3. Facts: Factual information about HPV and HPV vaccination 4. User involvement: Content engaging parents to e.g., share positive statements about HPV vaccination 5. Vaccine support: Stories about organizations or other groups showing their support of HPV vaccination 6. Events: Information about events related to HPV vaccination
Gerend et al. [40]	Country: USA Targeted group: college students Sample size: n/a Vaccine: HPV Intervention type: student-directed campaign materials promoting HPV vaccination (e.g., posters, yard signs, social media posts) Social media type: nonspecific Theory: None	To evaluate the effects of a human papillomavirus (HPV) vaccination intervention implemented on a large university campus in the United States.	A brief, relatively low-cost intervention was effective in increasing HPV vaccine administration among young adult students attending a large university in the United States. During the semester the intervention was implemented we observed more than a 75% increase in HPV vaccine doses administered at the university health center. The percent increase was similar for both the full sample and the sample restricted to ages 18–26 years. Although intervention	The intervention consisted of two primary components: (1) student-directed campaign materials and (2) provider-directed training with encouragement to recommend HPV vaccine to all eligible students. Content for the intervention was informed by theory and previous research and was designed to address common barriers and facilitators to HPV vaccination observed among young adults (e.g., misconceptions about HPV vaccination;
			materials emphasized the benefits of HPV vaccination for both men and women, fewer doses were administered to males than females in both years. Men are less likely than women to seek preventive health care services and thus may have fewer opportunities to receive the HPV vaccine. Indeed, most female students received the HPV vaccine from the UHS ‘‘women’s clinic.”	low perceived vulnerability to HPV infection; receiving a recommendation for HPV vaccination from a health care provider). Student-directed materials included yard signs posted across campus, posters distributed to the residence halls that changed every month of the intervention, a large banner hung in the UHS atrium, an HPV Questions & Answers page posted on the UHS website, and weekly social media posts.
La Torre et al. [42]	Country: Italy Sample size: 743 users Targeted group: online users Vaccine: General Intervention type: Facebook posts Social media type: Facebook Theory: None	To present the results of one year of this activity in terms of how the contents of the page were received, liked, and shared among its followers.	Events were the most popular type of news, followed by press releases, and scientific publications. Institutional Videos and documents are forms of communication less considered or appreciated by users. The day of the week in which users were most likely to be attracted by the contents of the links was Friday. Shares: Press releases were the communication form most shared by Facebook users, followed by scientific publications and institutional documents. No sharing of video links. Users shared more links on Fridays.	Facebook messages developed to share information about vaccination. Information materials chosen by health professionals and scientific communication experts published three times a week. Short and regular messages with breaks of one day approximately between publications. News with images were chosen more often.
Pedersen et al. [35]	Country: Demark Sample size: 8,020,000 people with an average of 127 comments per post Targeted group: Danish girls Vaccine: HPV Intervention type: A comprehensive social media strategy using ‘heart-brain communication Social media type: Facebook Theory: None	To evaluate whether the social media strategy developed for the campaign Stop HPV Stop cervical cancer was successful at engaging target groups in communication regarding HPV vaccination.	A comprehensive social media strategy using ‘heart-brain communication’ proved useful in a campaign for HPV vaccination. The success of the social media strategy was due to meticulous planning prior to launching the campaign, the use of content subgroups, the allocation of adequate resources for community management, the empirical analysis of content, and the use of evaluation results as guidance to produce new content.	‘Heart-brain communication’ combined facts and emotions through varied content. Community management guidelines were worked out to ensure that there was positive dialogue. Key Point Indicators (KPI) for Engagement Rate (ER) and Click Through Rate (CTR) were chosen to uphold engagement and traffic from Facebook to the website. The KPIs were used to measure effectiveness.
Robichaud et al. [30]	Country: Canada Target population: Medical students Sample size: *N* = 41 Vaccine: Influenza Intervention type: information sharing Social media type: YouTube Theory: None	Examine the prior attitudes of first year medical students to seasonal influenza immunization (their risk-benefit calculation, their sense of vulnerability to seasonal influenza, their overall attitudes towards immunization and their immunization history) and assess the impact of the most popular vaccine-critical YouTube videos on their attitudes towards seasonal influenza vaccine	The study did not find a significant difference in the responses to the questions asked before and after watching the videos.	This study randomly assigned medical students to watch one of two YouTube videos with different rhetorical styles (evidence- based versus anecdotal) and measured any change in attitudes and behaviors before and after watching the video
Allen et al. [25]	Country: USA Targeted group: women ages 18–26 years residing in low-come, public housing Sample size: 35 women Vaccine: HPV Intervention type: Twitter campaign Social Media type: Twitter Theory: The Health Belief Model	This study assessed the feasibility of implementing a one-month Twitter campaign to promote knowledge about the human papillomavirus (HPV) vaccine among low-income women living in public housing.	Most believed that Twitter was an acceptable educational strategy and remained engaged with the campaign throughout the intervention. It observed no changes in HPV knowledge, perceived benefits of or barriers to vaccination, decision self-efficacy, or vaccine intentions after the campaign, although perceived risk for cervical cancer decreased.	Women received a daily tweet over a period of one-month that contained messages from educational materials produced by the Centers for Disease Control and Prevention, National Cancer Institute, and the Massachusetts Department of Public Health. Messages were selected to align with the Health Belief Model and primarily addressed HPV vaccination, although there were also messages promoting cervical cancer screening.
Ortiz et al. [51]	Country: USA Sample size: adolescents (*N* = 38) Targeted group: Adolescents aged 13–18 Vaccine: HPV Intervention type: educational information Social media type: Facebook Theory: Health Belief Model	This study describes the formative research, execution, and evaluation of a social media health intervention to improve adolescents’ knowledge about and vaccination against human papillomavirus (HPV)	Adolescents are generally interested in receiving information about HPV and the vaccine, along with other relevant health information, through social media channels if messages are considered interesting, their privacy is protected, and the source is credible. Knowledge: Participants who reported receiving notifications for each new Facebook post were significantly more likely to have an increase in their HPV and vaccine knowledge but not in their vaccination rates.	A Facebook page, “About your Health”, with information about HPV vaccination and notifications received each time a new message was posted on the page. Maintained by local healthcare providers. 24 health facts (11 about HPV) were posted throughout a three-month period (with images and links to credible websites). Topics included virus susceptibility, virus severity, vaccine benefits, vaccine barriers and self-efficacy.
Lee and Cho [30]	Country: USA Sample size: 142 Target population: college students Vaccine: HPV Intervention type: information sharing Social media type: Facebook Theory: Health Belief Model	Investigate whether using different message framing and media influences the public’s perceived severity, benefits, barriers and willingness to get vaccinated.	Perceived severity of HPV: Participants who viewed the Facebook page perceived a lower severity than those who saw the online newspaper (p<0.05) Perceived benefits of getting vaccinated: No statistically significant medium effect between newspaper and Facebook (*p* = .94) Perceived barriers to getting vaccinated: Participants who viewed the Facebook page perceived lower barriers than those who saw the newspaper (*p* < .05) Willingness to get vaccinated: Participants who viewed the loss-framed message on Facebook mostly exhibited a higher level of behavioral intention to get vaccinated than those in the gain-framing condition (*p* < .05).	Participants exposed to four scenarios: 1. a gain-framed message on a fake look-alike Facebook page, 2. a loss-framed message on a fake look-alike Facebook page; or 3. a gain-framed message on a fake look-alike New York Times website or a 4. loss-framed message on a fake look-alike New York Times website. Gain-framed message: health benefits from getting the HPV vaccine (e.g., prevention of cervical cancer or genital warts) Loss-framed message: negative consequences of not getting the HPV vaccine (e.g. getting cervical cancer or genital warts).
Sundstrom et al. [31]	Country: USA Target population: university female students Sample size: *N* = 18 Vaccine: HPV Intervention type: information sharing Social media type: Facebook, Twitter, and Vine Theory: Health Belief Model	Describe the development, implementation, and evaluation of a theory-based cervical cancer prevention communication campaign for college-age women.	Uptake of HPV vaccination: Despite widespread coverage in the media, the messaging does not seem to have effectively changed behavior given the limited knowledge seen in focus groups. Among participants, 63% had heard of the campaign. Following the campaign, 93% had heard of the HPV vaccine and 74% believed the HPV vaccine was successful at preventing cervical cancer	Messages communicated about perceived threats, benefits, and safety of HPV vaccine. The main campaign message, “It’s my time”, encouraged the consideration of HPV vaccination and regular screening, and reminded individuals that it is not too late to receive the HPV vaccine. Messages were delivered through mass media and social media (Facebook, Twitter, and Vine). Twitter and Facebook were updated daily with relevant news articles, pictures and facts and messages included the hashtag #MyTime. A video contest was also prepared on Vine, where participants had to submit a video that finished the sentence: “I received the HPV vaccine so that I have time to…”
Mohanty et al. [41]	Country: USA Sample size: 155,110 adolescents and engaged 2106 adolescents Targeted group: adolescents Vaccine: HPV Intervention type: Facebook campaign; Reminder-recall letters Social media type: Facebook Theory: Health Belief Model	To use Facebook to reach adolescents for human papillomavirus (HPV) vaccination	The campaign was well-received, far-reaching, and generated awareness and conversations among adolescents. However, the campaign did not appear to be a sufficient driver for HPV uptake even when common barriers to HPV immunization were minimized. Uptake of HPV vaccination: On average, each advertising campaign reached 155,110 adolescents and engaged 2106 adolescents. The advertising campaigns that focused on HPV disease risk and local resources were the most successful in engaging adolescents. Overall, 3400 adolescents became fans of the campaign and 176 doses of HPV vaccine were administered to 152 adolescents, out of which 63 received the three doses.	Between July 2012 and July 2013, adolescents with a Facebook account who self-identified as being between the ages of 13–18 years old and living in Philadelphia were targeted to receive a series of advertisements from 3forME website. The website had multiple tabs, which included information about: (1) HPV vaccine and disease, (2) the 3forME campaign, (3) a sign-up page for adolescents to receive their HPV vaccine at a local clinic or a community event and, (4) an upcoming events page which featured dates and times where the 3forME team would be administering HPV vaccine. Content for the campaign was adapted from the Centers for Disease Control and Prevention to ensure cultural sensitivity and a readability level appropriate for adolescents.
Brandt et al. [38]	Country: USA Sample size: two undergraduate classes (*n* = 58) Targeted group: undergraduates Vaccine: HPV Intervention type: technology-mediated, social media-based intervention Social media type: Facebook Theory: Health Belief Model	To evaluate a novel, technology-mediated, social media-based intervention to promote HPV vaccination among college students	Findings suggest the approach was effective in improving knowledge about HPV and HPV vaccination. Participants reported that the intervention addressed gaps in knowledge related to men’s susceptibility to HPV and the link between HPV and oral cancer. Participants also described how increased knowledge led to improved confidence and inspired conversations about HPV vaccination with family and friends. At the start of the 9-week study, approximately half of participants had previously received the full HPV vaccination series (57%), which represents a considerably higher rate for full vaccination coverage than might have been expected based on estimates for adolescents in South Carolina (43%). At the conclusion of the HPV intervention, the number of participants initiating/completing the vaccination series increased, as did the number of young adults indicating they intended to get vaccinated in the next 6 months.	Messages were tailored to the campus and local area, with resources for how and where to obtain the HPV vaccination at low or no cost, including the university’s Student Health Services. Study investigators moderated the Facebook page, which included providing answers to questions and stimulating interaction, as well as sharing timely news articles and studies related to HPV vaccination. The posts were designed to cultivate a social climate of support for considering initiation of the vaccination series (or completion of the series for those who had partial completion of the sequence). Polls provided social norming to stimulate vaccination-related discussion. Intervention activities (e.g., reading the newsletters and interacting on Facebook) were intended to require approximately 30 total minutes per week. The program spanned a 9-week period during the spring semester, with a hiatus during spring break week.
Dialogue-based				
Haase et al.[26]	Country: Germany Target population: online users Sample size: 265 participants Vaccine: general Intervention type: information sharing Social media type: Online forum Theory: None	Assess the potential moderating effect of statistic and narrative source credibility on the biasing effect of narrative information regarding the perception of vaccination risks	Researchers found that narratives discussing vaccine adverse events decreased intentions to get vaccinated and increased perceptions of vaccination risk. This bias occurred irrespective of whether the post was read on the neutral online health forum or the anti-vaccination website.	Researchers aimed to understand if the credibility of the source of a forum post influenced the readers’ perception of vaccination risk. They did this by presenting identical narratives, but with different introductory texts (one from a neutral online health forum and the other from a known anti-vaccination website).
Langley et al. [29]*	Country: Netherlands Target population: parents of daughters who would be invited to get their HPV vaccination Sample size: 184 parents Vaccine: HPV Intervention type: Online group discussions Social media type: Online discussion forums Theory: Health Belief Model	Develop knowledge about whether and which interventions can be used online to actively support offline vaccination behavior once negative information has been spread via online social media.	We find that opinions relating to the vaccination within the nuclear family have the strongest relationships, suggesting that influences via OSM may need to concentrate not just on one decision-maker, but on the interdependent family members. The parent’s opinion about the vaccination (valence) is less strongly related to that of the daughter, but whether they agree or not, the daughter’s opinion is highly influential on the decision whether to vaccinate. The partner’s opinion, on the other hand, predominantly influences the participant’s own opinion (valence). In contrast, parents’ friends and peers have a far weaker effect on the decision to vaccinate, whereby there appears to be almost no difference in influence between a person’s close friends, the other parents they know in their social environment, and people they interact with via OSM.	Parents took part in discussion forums, where they were exposed to the following conditions: Participants randomly assigned to the conditions of a 2 (source peer vs governmental organization) x4 (influence strategy: source credibility, self-belief, direct challenge, indirect challenge) between-subjects design. Online discussion group to discuss raising adolescents and related issues. Parents were asked to log in on a specified date and time and to be available to participate in the experiment for 50 minutes. Some messages were sent by the study team to look like they came from peers or government officials and were followed by a pro-vaccination comment with different persuasion strategies.
Lai et al. [28]	Country: China Sample size: 200 female adolescents Targeted group: female adolescents Vaccine: HPV Intervention type: Facebook-assisted online discussion Social media type: Facebook Theory: Health belief model	To identify the effectiveness of a Facebook-assisted teaching method on female adolescents’ knowledge and attitudes about cervical cancer prevention and on their human papillomavirus vaccination intention.	Knowledge, personal attitude, and behavioral intention: Under the condition of the vaccine being an out-of-pocket expense, students receiving a Facebook-assisted teaching method were 1.810 (measured two weeks after the Facebook teaching method) and 1.847 (measured eight weeks after the Facebook teaching method) times more likely to have the intention to be vaccinated compared with students who received traditional teaching instruction. Under the condition of receiving the vaccine free of charge, this figure was 2.531 times higher. A comparison of change across groups indicates that knowledge improvement scores in the experimental group were 2.942 points greater than those in the control group. Attitude improvement scores in the experimental group were 3.888 points greater than those in the control group.	Cervical cancer prevention education (CCPE) programme based on the Health Belief Model, developed by Ministry of Health to assist teens in understanding cervical cancer, Pap Smear screening and HPV vaccination and cervical cancer prevention The lecturer, a nursing teacher, initiated the discussion with the question: What is an HPV vaccine? This was followed by a brief introduction of the HPV vaccine and the CCPE lecture commenced. The lecture concluded with a brief summary and discussion, reflecting on the importance of cervical cancer prevention. Six-hour discussion sessions were offered either with Facebook-assisted or in-person discussions after class.
Liao et al. [31]	Country: China Sample size: 365 mothers Targeted group: Young Children Vaccine: Influenza Intervention type: online discussion groups Social media type: social networking apps such as WhatsApp Messenger Theory: Social Cognitive Theory	To test the effectiveness and parental acceptability of a social networking intervention that sends weekly vaccination reminders and encourages exchange of SIV-related views and experiences among mothers via WhatsApp discussion groups for promoting childhood SIV.	Online information support can effectively promote mothers’ self-efficacy for taking children for SIV but alone it may not sufficient to address maternal concerns over SIV to achieve a positive vaccination decision. Results showed that there were significant intervention effects on the change of participants perceived self-efficacy in taking children for SIV, with participants of the SNI–TP and SNI+TP groups reporting more increase in confidence in taking their children for SIV than did the control participants. The most common participant discussion topics were vaccination decisions followed by vaccination clinic and cost, vaccine safety and side effects, and vaccine effectiveness	Vaccination Reminders: Message 1 introduced the CIVSS and doctors’ recommendations for children’s SIV, message 2 addressed children’s risk of seasonal influenza and benefits and safety of SIV for children, and message 3 addressed the number of days remaining for the recommended vaccination timing; WhatsApp Discussion Groups: In each WhatsApp discussion group, mothers could post their opinions and concerns about influenza and SIV and freely communicate with other mothers and the group moderator about their experiences of personal and child influenza vaccinations
Kimmerle et al. [27]	Country: Germany (although unclear whether the forums are German) Target population: The users of 28 various Internet forums Sample size: n/a Vaccine: Measles Intervention type: Online group discussions Social media type: Internet Forum	Examine in what way the particular type of contribution (i.e., factual information vs personal experiences) has an impact on the subsequent communication in health-related Internet forums.	There was no statistically significant increase in the number of responses on the personal experience posts as compared to the factual post. However, responses to the personal experience post were significantly more emotional than responses to the factual information post.	This study compared the differences between using factual information versus personal experience in a post on health-related Internet forums. The researchers observed how each condition of the initial post affected subsequent communication on the forum. They considered the number of responses each post received and how emotional the language/content of the posts were.
**Interactive website**				
Shoup et al. [37]	Country: USA Sample size: *N* = 443 Target population: pregnant mothers and parents of children younger than 4 years Vaccine: General Intervention type: Interactive website Social media type: Website with built-in interactive component Theory: the theory of planned behavior and the Health Belief Model	Describe a process for designing, building, and evaluating a theory-driven social media intervention tool to help reduce parental concerns about vaccination.	Survey results suggested that social media may represent an effective intervention tool to help parents make informed decisions about vaccination for their children. Approximately 50% of parents in all three vaccine behavior groups reported that they would use the web-based tool often. More than 60% of parents who delay or accept vaccines reported that they would trust the information about vaccines presented on the tool. Regardless of their vaccine decisions, a high proportion of parents reported that they would use the tool to ask questions, to receive current vaccine information, and to review the childhood vaccination schedule.	An interactive web-based tool that provides evidence-based information in an interactive environment where parents can contribute content and discuss concerns with other parents and vaccine experts. Authors developed, pilot-tested, and mailed a survey to assess their hypothetical trust in and use of a social media web-based tool for vaccine and health information. A manual medical record review was then conducted on the children to determine if parents had delayed or refused vaccination for personal, nonmedical reasons.
Daley et al. [39]	Country: USA Sample size: 1,093 participants Targeted group: Parents of infants or pregnant Vaccine: General Intervention type: multi-components Social media type: Website with built-in interactive components Theory: the Health Belief Model and the Theory of Planned Behavior	To assess whether an Internet-based platform with vaccine information and interactive social media components improved parents’ vaccine-related attitudes.	Among 542 participants in the VSM study arm, 189 (35%) visited the study website at least once, with a mean of 1.9 visits (*SD* = 1.8) and a range of one to 15 visits. Personal attitude and behavioral intention: Interventions were associated with significant improvements in attitudes regarding vaccination benefits compared to usual care among vaccine-hesitant parents. Interventions were associated with significant reductions in parental concerns about vaccination risks compared to usual care among hesitant parents. Perceived self-efficacy also improved,	Internet-based platform with vaccine information and interactive social media components 3 arms: 1- vaccine social media (VSM) arm, access to website with vaccine information and interactive social media components; 2 Vaccine information (VI) arm (website without social media), and 3- usual care (UC) arm Social media format: blog, discussion forum, chat room. New blogs posts added by the research team every month covering timely or controversial issues; Ask a question portal available as well to direct questions to experts-responses
			although a significant change was only observed when comparing VI. No significant differences were observed when comparing the VSM versus VI study arms. Change in attitudes over time among parents who were not vaccine hesitant at baseline: The VSM and VI interventions were not associated with any significant changes in vaccine-related attitudes compared to usual care	provided within 2 days. Online chat sessions held each month to engage in conversations with vaccine experts and between participants. Monthly newsletters to encourage website use.
Glanz et al. [41]	Country: USA Sample size: 888 parents Targeted group: parents of infants Vaccine: infant vaccine Intervention type: Web-based social media with vaccine information and interactive social media components (VSM) Social media type: n/a Theory: Health Belief Model and Theory of Planned Behavior	To determine if a Web-based, social media intervention increases early childhood immunization.	This RCT of a Web-based vaccine on early childhood immunization. The intervention had a positive impact information and social media Pregnant women exposed to the VSM arm were more likely to vaccinate their infants on time than participants receiving UC, these results suggest that interactive, informational interventions administered outside of the physician’s office can improve vaccine acceptance	A 3-arm, randomized controlled trial was conducted in Colorado from September 2013 to July 2016. Participants were pregnant women, randomly assigned (3:2:1) to a website with vaccine information and interactive social media components (VSM), a Web site with vaccine information (VI), or usual care (UC). Vaccination was assessed in infants of participants from birth to age 200 days. The primary outcome was days under vaccinated, measured as a continuous and dichotomous variable.
Personal reminder				
Patel et al. [34]	Country: USA Sample size: 365 women Targeted group: women aged 19–26 Vaccine: HPV Intervention type: Reminder system (-text, e-mail, phone, private Facebook message, or standard mail) Social media type: Facebook Theory: None	To evaluate whether automated reminders increase on-time completion of the three-dose human papillomavirus (HPV) vaccine series.	The study intervention did not increase HPV vaccine series completion. Despite great public health interest in HPV vaccine completion and reminder technologies, completion rates remain low.	At the time of the enrollment visit, women in the intervention arm also selected their preferred method for reminders – text message, e-mail, phone call, private Facebook message, or standard mail. The “Staying on Track” software system, designed for this study, recorded subject data, and sent the automated reminders. Each intervention participant received four messages (one if she selected standard mail), sent three days apart prior to doses two and three. The reminder schedule mirrored the recommended dosing schedule. Reminders for dose two were sent six weeks after the initial visit. Timing of reminders for dose three was dependent on when the participant returned for dose two; reminders were sent either 12 weeks after the second dose or 24 weeks after the first dose, whichever was sooner.
